# The relevance of chest X-ray radiologic severity index and CURB-65 score with the death event in hospitalized patient with COVID-19 pneumonia

**DOI:** 10.1186/s43055-022-00877-y

**Published:** 2022-08-25

**Authors:** Nicolaus Erlangga Prasetyo, Bambang Satoto, Thomas Handoyo

**Affiliations:** Faculty of Medicine, Universitas Diponegoro – Doctor Kariadi General Hospital, Semarang, Indonesia

**Keywords:** COVID-19 pneumonia, CURB-65, Chest X-ray, Radiologic severity index, Mortality

## Abstract

**Background:**

The global pandemic respiratory infection COVID-19 has had a high mortality rate since early 2020 with a broad spectrum of symptoms and giving a high burden. This study used the chest X-ray radiologic severity index method to radiologically assess the degree of lung lesions and the CURB-65 score to clinically assess COVID-19 pneumonia patients associated with the incidence of death in hospitalized patients.

**Results:**

The research data were carried out from March 2020 to April 2021 based on patient medical records and chest X-rays at Doctor Kariadi General Hospital Semarang Indonesia. One hundred and five subjects were collected that fulfilled the inclusion and exclusion criteria. The CURB-65 score ≥ 2 had a significant relationship to the death event with a prevalence interval of 2.98 (95% CI, *p* = 0.000). The radiologic severity index ≥ 22.5 in initial chest X-ray signified a prevalence ratio of 2.24 (CI 95%, *p* = 0.004) and the radiologic severity index ≥ 29.5 in the second chest X-ray signified a prevalence ratio of 4.53 for the incidence of death (95% CI, *p* = 0.000). The combination of CURB-65 and the first chest X-ray radiologic severity index resulted in a prevalence ratio of 27.44, and the combination of CURB-65 and the second chest X-ray radiologic severity index resulted in a prevalence ratio of 60.2 which were significant for the mortality of hospitalized COVID-19 pneumonia patients.

**Conclusions:**

Chest X-ray radiologic severity index and CURB-65 score have a significant relevance with the death event in hospitalized patients with COVID-19 pneumonia and can thus be used as a predictor of mortality.

## Background

Coronavirus disease 2019, or better known as COVID-19, is a disease with clinical manifestations of pneumonia which was first discovered in Wuhan City, Hubei Province, China, at the end of December 2019. The disease is caused by severe acute respiratory syndrome coronavirus-2 (SARS-CoV-2) and is spreading rapidly and globally. On 12 March 2020, the World Health Organization declared the global pandemic status of COVID-19 [1–3].

The diagnosis of COVID-19 was made based on a combination of anamneses and contact history, physical examination, haematological examination, and imaging. Imaging is used as a feasible screening modality to detect early pneumonia features and provide an overview of the severity, disease progression, and post-infection control. Using radiologic severity index (RSI) as a method of assessing X-ray lesions provides an index of thoracic lesion values based on two parameters: the percentage of lesion area and the degree of lesion density. The CURB-65 score is widely used as a clinical screening to predict the need for intensive care and the incidence of death in community-acquired pneumonia [1, 4, 5].

This study aimed to obtain the relevance between radiological features based on RSI of chest X-ray and clinical assessment based on CURB-65 score with the incidence of death in COVID-19 pneumonia patients. The results of this study are expected to be used as a basis for management and predictors of mortality in COVID-19 pneumonia.

## Methods

This study is a diagnostic test using a cross-sectional method with a retrospective approach. The research data were carried out from March 2020 to April 2021 based on patient medical records and chest X-rays at Doctor Kariadi General Hospital Semarang. The research subjects were male and female patients aged 18 years or older who were tested positive for COVID-19 and received inpatient services at the Doctor Kariadi General Hospital Semarang. The minimum sample size of this study was determined based on the prospective test formula to get the relative risk index. The formula *np* = *Zα*^2^ (*Q*1/*P*1 + *Q*2/*P*2)/(ln (1−*e*)^2^), with a significance level of 0.05, CI 95%, and power of 80%, according to the incidence of death due to COVID-19 in Indonesia of 4.1% (on September 8, 2020) to get a minimum sample size of 80.3 samples.

The inclusion criteria in this study were COVID-19 inpatients and received serial chest X-ray examination services at least 2 times in 1 hospitalization period. Exclusion criteria for study subjects were the presence of comorbid diseases or a history of previous chest X-ray images that could resemble COVID-19 pneumonia or could obscure the assessment of pulmonary lesions, including a history of pulmonary tuberculosis, history of lung malignancy or lung metastases, pulmonary oedema, history of interstitial lung disease, pleural effusion, pleural mass, or chest wall mass. Although more than 5000 sample candidates have been obtained, there were only a little less than 200 samples that met the inclusion and exclusion criteria. Moreover, many samples did not have a complete data set.

### The CURB-65 score

**Fig. 1:**
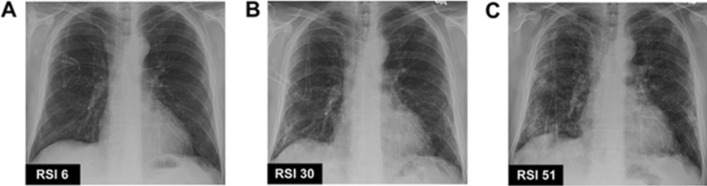
Representative radiologic severity index score serial for chest X-ray. RSI scores are labelled within each panel. Panels **A–C** show chest X-ray images from an individual patient with parainfluenza virus-associated lower respiratory infections from the previous study in America, in order of increasing severity [4]

The CURB-65 score was assessed based on the initial general condition and vital signs when the patient was admitted, as well as the creatinine value of the first blood examination. The CURB-65 assessment is based on the following factors: level of consciousness (confusion), blood urea (uremic), respiratory rate (respiratory), blood pressure (blood pressure), and age (≥ 65). Consciousness is assessed whether the patient has decreased consciousness or not. Blood urea nitrogen levels are declared to be valuable if they are more than 20 mg/dl. The respiratory rate was assessed if it was more than 30 breaths per minute. Blood pressure is declared at risk if the systolic blood pressure is less than 90 mmHg or the diastolic blood pressure is less than 60 mmHg. The risk factor for age is worth if it is more than equal to 65 years. Each criterion obtained is worth 1, so the total score ranges from 0 to 5 [5–7] (Fig. 1).

### Chest X-ray radiologic severity index

**Fig. 2:**
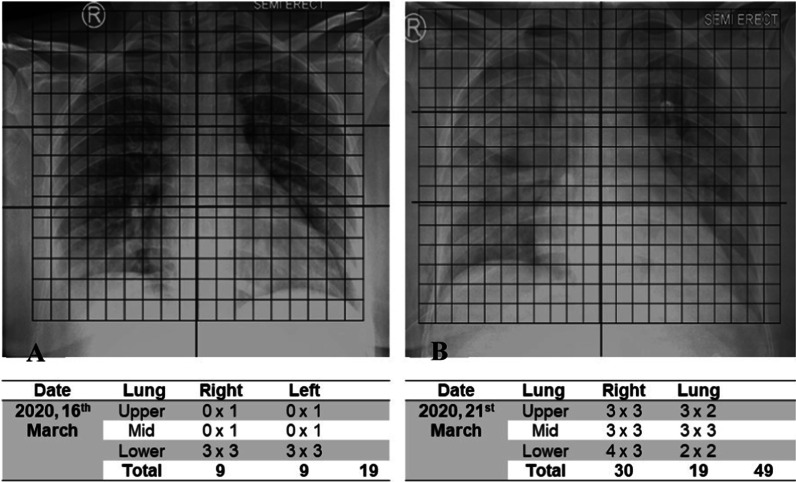
Radiologic severity index assessment in COVID-19 pneumonia patient. This image shows serial chest X-ray RSI values in same patient with COVID-19 pneumonia. **A** RSI values on initial chest X-rays at 16 March 2020 and **B** RSI values on serial chest x-rays at 21 March 2020. There is an increase in RSI value at 5-day interval

This study uses a posteroanterior or anteroposterior projection chest X-ray. The entire chest X-ray sample was assessed using the RSI method. The RSI scoring system uses two main variables: the pattern of spread and the volumetric density of the lesion. In the RSI assessment, the spread of the lesion was assessed based on the location of the lesion in the lung fields, where the right and left lung fields were divided into three parts, respectively; the upper zone (up to the carina), the middle zone (below the carina to the upper border of the inferior pulmonary vein), and the lower zone (below the upper border of the inferior pulmonary vein). An inferior pulmonary vein can be identified by a linear image of the right hilum pointing to the periphery, crossing the pulmonary artery [8]. The lesion pattern was divided into 3 categories; 1 for normal lungs, 2 for ground-glass opacity images, and 3 for consolidation images. The volumetric area is divided into 5 broad categories; 0%, 1 for 1–24% lesion area, 2 for 25–49% lesion area, 3 for 50–74% lesion area, and 4 for 75–100% lesion area. The RSI was obtained by adding up the multiplication of the value of the lesion pattern and its volumetric area in each zone, with a total value between 0–72. The chest X-ray RSI assessment of the study is shown in Fig. 2. The independent variable of this study was the factors that formed the values of RSI and CURB-65. The dependent variable in this study was the RSI chest X-ray, the CURB-65 score, and the incidence of death in hospitalized patients with COVID-19 pneumonia [4].

### Statistical analysis

A receiver operating characteristic (ROC) curve was used to determine the cut-off value with high sensitivity and specificity in the research. Furthermore, an analysis of the area under the curve (AUC) was carried out to measure the accuracy of the diagnostic test. Cross tabulation of the cut-off values of RSI and CURB-65 was analysed using the chi-square method with the results of a significant relationship analysis if *p* value < 0.05 and continued with the analysis of prevalence ratio. Hypotheses testing was continued by using the logistic regression method to obtain a multivariable relationship between CURB-65 and RSI as a predictor of mortality.

## Results

**Fig. 3:**
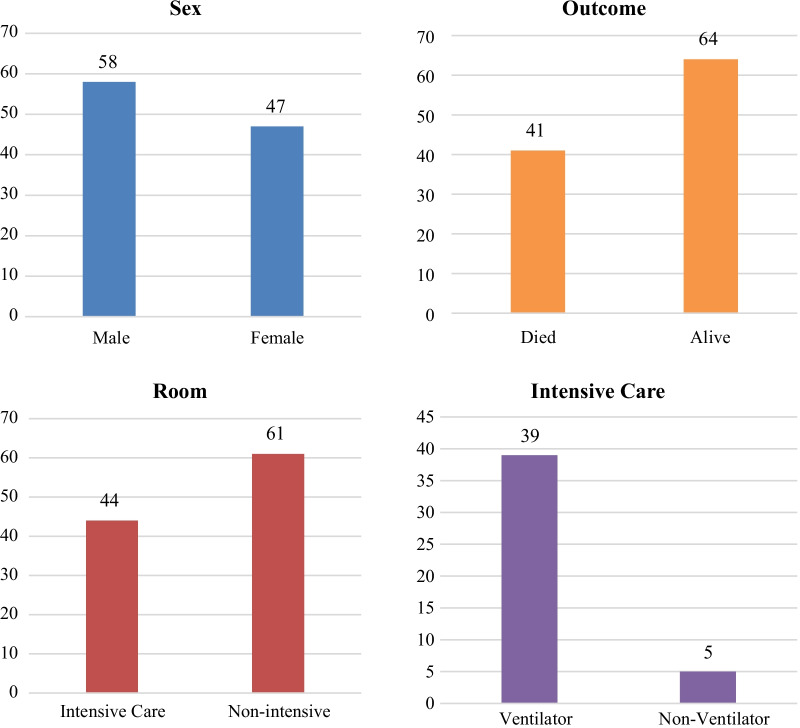
Characteristic of hospitalized COVID-19 pneumonia patients. The graph shows the characteristics of COVID-19 pneumonia patients based on sex, post-treatment outcome, intensive room, and ventilator usage

In this study, 105 research subjects were collected, consisting of 58 men (55%) and 47 women (45%). The mean age of the subjects was 51.3 with the youngest being 23 years old, the oldest being 77 years old, and the median age was 54 years old. The average day of hospitalization was 13.5 days, with the shortest being 1 day and the longest being 43 days. During the hospitalization period, 61 subjects received inpatient services in the non-intensive room (58.1%) and 44 subjects were treated in the intensive room (41.9%). Of the 44 patients treated in the intensive care unit, 39 patients were treated using a ventilator (37% of the total subjects). A total of 41 subjects were declared dead at the end of the treatment period (39.1%) and 64 subjects were declared cured and could be outpatient (60.9%). During treatment, patients receive serial X-ray examination services to support the diagnosis with an average of 3.2 photos during a hospitalization period. The minimum number of serial photos is 2 photos and the maximal number of serial photos is 9 photos per subject in 1 hospitalization period. Furthermore, an analysis test was carried out on 104 subjects because it was found that 1 subject had missing data. The characteristic of the research subjects is shown in Fig. 3.

The first hypothesis test was conducted to obtain the relationship between the CURB-65 value and post-treatment outcomes. This study used CURB-65 ≥ 2 as cut-off value, according to the study of Satici, Asim, Sargin, et al. to estimate the risk of death of the subjects [6]. From the sample of this study, 19 subjects with a CURB-65 ≥ 2 were found and 16 of them died (84.2%). A total of 85 subjects with a CURB-65 value < 2, 24 subjects were declared dead (28.2%). The chi-square method was used to test the relationship between the value of CURB-65 with post-treatment outcomes. From the test results, there is a significant relationship between the value of CURB-65 and post-treatment outcomes. Both subjects died and recovered with *p* = 0.000 (significant if *p* < 0.05; 95% CI). The cut-off value of CURB-65 ≥ 2 gave a sensitivity of 40%, specificity of 95%, a positive predictive value of 84%, and a negative predictive value of 71%. The cut-off value of CURB-65 ≥ 2 on the incidence of death gives the result of prevalence ratio (PR) of 2,982. This PR value indicates that subjects with a CURB-65 ≥ 2 have a 2,982 times higher probability of death than subjects with a CURB-65 value of < 2. This result is given in Table 1.

**Table 1:** Cross-tabulation of CURB-65 category to outcome

CURB-65 category	Outcome		*p*	PR (95% CI)
	Died	Alive		
≥ 2 (*n* = 19)	16 (84.2%)	3 (15.8%)	0.000	2.982 (2.018–4.409)
≤ 1 (*n* = 85)	24 (28.2%)	61 (71.8%)		

*PR* prevalence ratio

**Fig. 4:**
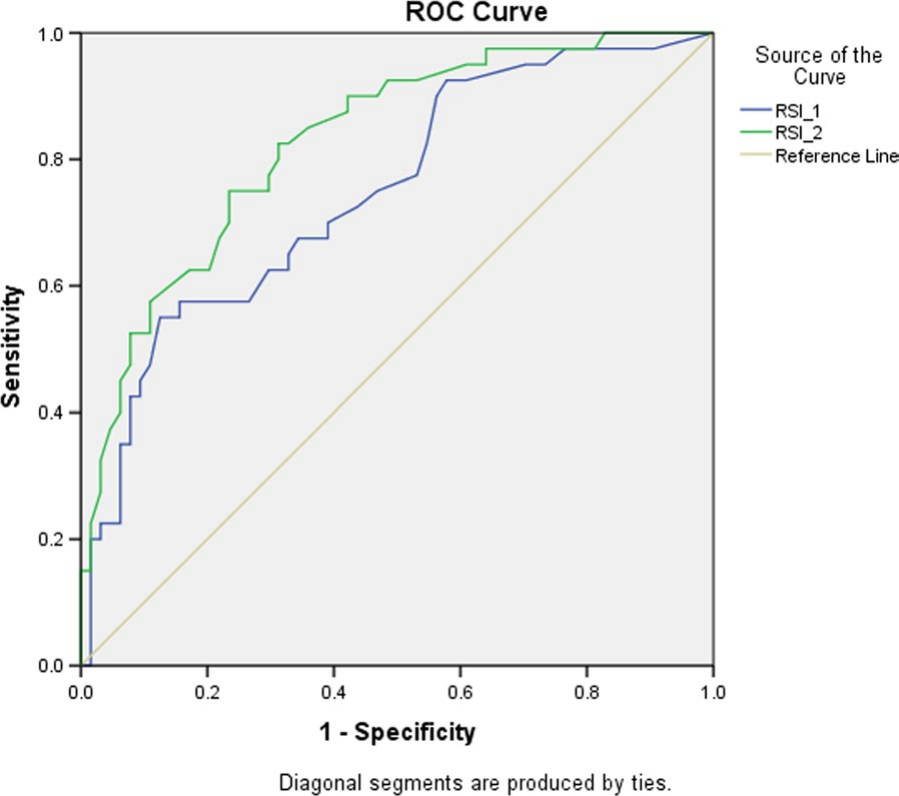
Receiver operating characteristic curve of RSI1 and RSI2. ROC curve showing the RS1 value curve (blue colour) and the RSI2 value curve (green colour) which are above the reference line. The curve of the RSI1 and RSI2 values has a fairly wide area under the curve and has good sensitivity as a measuring tool. From the figure, it can be seen that the area under the RSI2 curve is wider than the RSI1 curve

Data analysis was carried out on the initial chest X-ray RSI value (hereinafter referred to as RSI1) and the serial chest X-ray RSI value (hereinafter referred to as RSI2) because all research subjects had the data. The third chest X-ray and so on were not analysed because not all patients were given the same service for the third and so on. The mean RSI1 value in subjects who were declared dead was 35.2 (SD 17.2), while the average RSI1 value in subjects who were declared cured was 19.5 (SD 15.2). The mean RSI2 value for subjects who were declared dead was 44.9 (SD 15.1), while the mean RSI2 value for subjects who were declared dead was 23.6 (SD 15.9). The ROC curve is shown in Fig. 4 (Table 2).

**Table 2:** Description of chest X-ray RSI1 and RSI2 to outcome

	Outcome		Statistic	Std. error
RSI1	Died	Mean	35.28	2.735
		Median	36.00	
		Std. deviation	17.295	
		Minimum	0	
		Maximum	62	
	Alive	Mean	19.50	1.904
		Median	20.00	
		Std. deviation	15.233	
		Minimum	0	
		Maximum	66	
RSI2	Died	Mean	44.90	2.402
		Median	47.00	
		Std. deviation	15.189	
		Minimum	6	
		Maximum	72	
	Alive	Mean	23.66	1.998
		Median	21.00	
		Std. deviation	15.981	
		Minimum	0	
		Maximum	61	

RSI1: radiologic severity index of first chest X-ray

RSI2: radiologic severity index of second chest X-ray

Based on the data distribution of RSI1 values, hypothesis testing was carried out using a cut-off value of 22.5 in order to obtain a sensitivity result of 70% and a specificity of 60%. The cut-off value of 22.5 was tested using the chi-square method with the results that there was a significant relationship between the RSI1 value and the incidence of death *p* = 0.004 (significant *p* < 0.05; 95% CI). The risk estimation analysis resulted in a PR value of 2.245, as given in Table 3. These results indicate that subjects with RSI1 ≥ 22.5 have a 2.245 times greater probability of death than subjects with RSI1 < 22.5.

**Table 3:** Cross-tabulation RSI1 and RSI2 categories to outcome

	Outcome		*p*	PR (95% CI)
	Died	Alive		
*RSI1 category*				
≥ 22.5 (*n* = 53)	28 (52.8%)	25 (47.2%)	0.004	2.245 (1.287–3.916)
< 22.5 (*n* = 51)	12 (23.5%)	39 (76.5%)		
*RSI2 category*				
≥ 29.5 (*n* = 53)	33 (62.3%)	20 (37.7%)	0.000	4.536 (2.210–9.313)
< 29.5 (*n* = 51)	7 (13.7%)	44 (86.3%)		

*PR *prevalence ratio

**Fig. 5:**
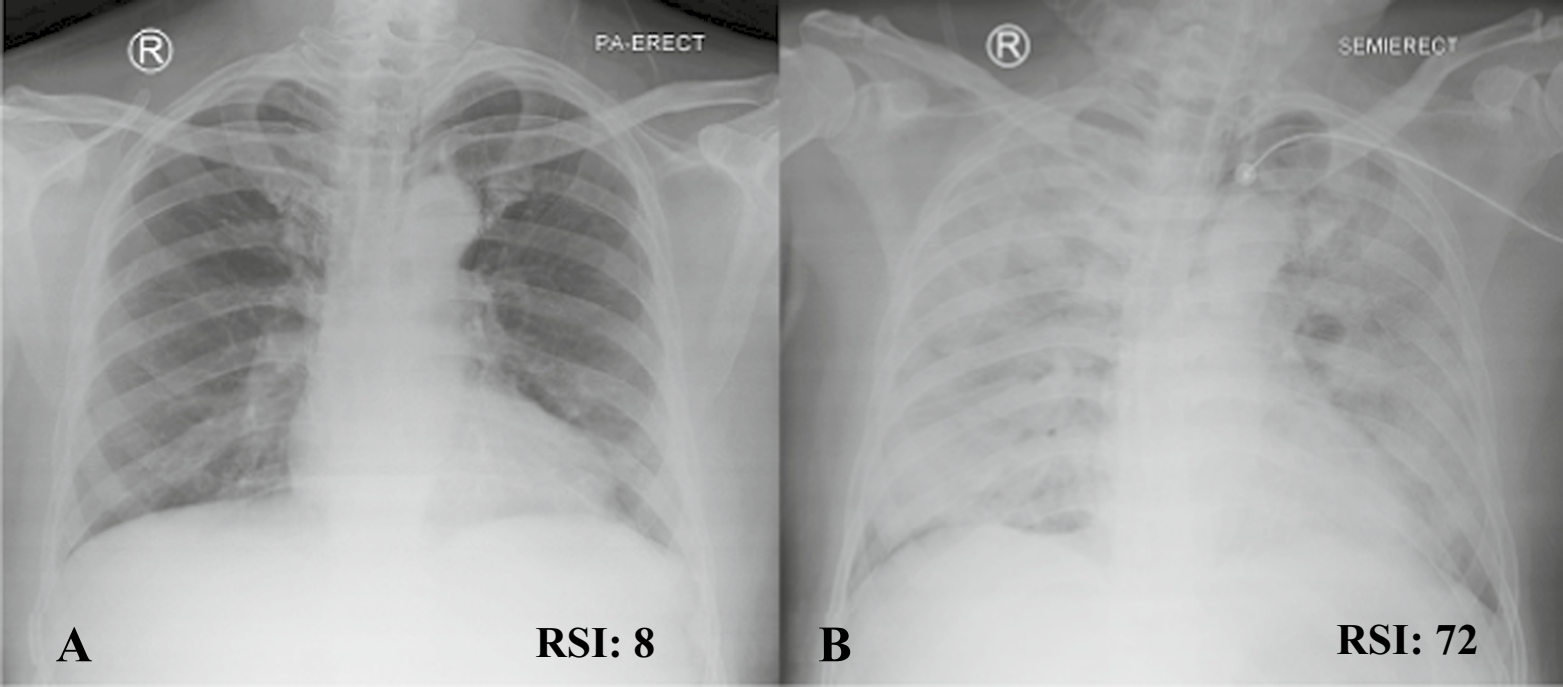
Serial radiologic severity index in COVID-19 pneumonia patient. **A** Initial chest X-ray RSI at 22 March 2020; **B** serial chest X-ray at 29 March 2020. An increase in the RSI value can be seen from an increase in the area and density of the lesion on the first and second chest X-rays

Based on the distribution of RSI2 data, a cut-off value of 29.5 was taken to obtain a sensitivity of 82% and a specificity of 68%. After testing the relationship using the chi-square method, it was found that there was a significant relationship between the RSI2 value and the incidence of death *p* = 0.000 (significant *p* < 0.05; 95% CI). The RSI2 risk estimation test with a cut-off value of RSI2 ≥ 29.5 resulted in a PR of 4.536, as given in Table 3 also. These results indicate that subjects with RSI2 ≥ 29.5 have a 4.536 times greater probability of death than subjects with RSI2 < 29.5 (Fig. 5).

A logistic regression test was conducted to obtain a predictive analysis of an outcome based on several variables. A logistic regression test was performed on a high CURB-65 value (cut-off value ≥ 2) and a high RSI1 value (cut-off value ≥ 22.5) on the incidence of death. Table 4 shows the logistic regression test of CURB-65 score, RSI1, RSI2, with the outcome. The logistic regression test in the high CURB-65 and RSI1 value categories gave a significant PR value of 27.44 with *p* = 0.000 (significant *p* < 0.05, 95% CI). Using the logistic regression test in the CURB-65 value category and high RSI2 gave a significant PR value of 60.2 with *p* = 0.000 (significant *p* < 0.05, 95% CI).

**Table 4:** Logistic regression test of CURB-65 + RSI1 and CURB-65 + RSI2

	B	S.E	Wald	df	Sig	Exp (B)	95% C.I. for EXP (B)	
							Lower	Upper
CURB + RSI1	3.312	0.845	15.357	1	.000	27.444	5.236	143.841
CURB + RSI2	4.098	0.891	21.129	1	.000	60.200	10.490	345.462

## Discussion

The results of the analysis showed that there was a significant relationship between the CURB-65 score on post-treatment outcomes for COVID-19 pneumonia patients. The CURB cut-off value of 2 was used to predict the mortality of hospitalized patients with COVID-19 pneumonia. The results of this relationship test are in accordance with previous research conducted by Rylance and Waitt, which showed that the value of CURB-65 can provide good results in the clinical assessment of pneumonia and determine the degree of severity [9]. These results are also in accordance with research conducted by Alavi-Moghaddam which showed that the accuracy of CURB-65 in predicting mortality includes the need for ICU in patients with community pneumonia such as COVID-19 pneumonia [7]. These results are also in agreement with the study of Satici, Asim, Sargin, et al. which showed that the cut-off value of CURB-65 ≥ 2 was significant in predicting the mortality of hospitalized patients with COVID-19 [6]. Although it has a lower sensitivity (40%) when compared to previous studies (75%), the CURB-65 value in this study provides greater specificity (95%) than previous studies (85%) in patients with COVID-19 pneumonia. This difference can be caused by differences in the number, inclusion and exclusion factors of research subjects, distribution of data, and different treatments. However, from the analysis test results, it was also obtained a PR value of 2.98 which indicates that subjects with a CURB value of 2 have a probability of death 2.98 times higher than those with a CURB value of < 2.

Hypothesis testing also showed a significant relationship between RSI values ​​and post-treatment outcomes for COVID-19 pneumonia patients. The cut-off value of RSI1 22.5 gives a PR of 2.245, which means that subjects with an RSI1 value of more than equal to 22.5 have a probability of death 2.245 times higher than subjects with an RSI1 value of less than 22.5. The PR RSI2 value of 4.536 indicates that subjects with an RSI2 value greater than 29.5 have a probability of death 4.536 times higher than subjects with an RSI2 value of less than 29.5.

The results of the RSI1 and RSI2 diagnostic tests are in line with previous studies conducted by Sheshadri, Shah, Godoy, et al., which found that the X-ray scoring system using the RSI method is associated with increased mortality [4]. RSI is able to show disease progression through changes in values ​​that describe changes in the density and area of ​​thoracic lesions. The area under the ROC curve is large, indicating that the RSI method can be used to evaluate chest radiographs based on their sensitivity and specificity. In accordance with the research conducted by Borghesi, Zigliani, Golemi, et al., and the research of Lotfi, Sefidbakht, Moghadami, et al., the results of the RSI value showed a significant correlation between mortality and the severity of COVID-19 [10–12].

This study also showed a multivariable relationship between CURB-65 and the RSI value for the incidence of death based on logistic regression test. The logistic regression test between the CURB-65 value and the RSI1 value showed significant results (*p* < 0.000) with a PR of 27.44. This value is higher than the single PR CURB-65 (2.982) and the single RSI1 value (2.245). Likewise, the results of the logistic regression test between the CURB-65 value and the RSI2 value showed significant results (*p* < 0.000) with a PR of 60.2. This value was also higher than the single PR CURB-65 and the single PR RSI2 (4.536). The test results indicate that there is a significant relationship between the CURB-65 value and the RSI value on the incidence of death. Although using a different severity assessment method from the previous research which used the Brixia system (Borghesi, Zigliani, Golemi, et al.), the results of the logistic regression test of this study also showed similar results. Patients with high chest radiography values,​accompanied by the presence of predictive factors, have a high risk of death in treatment [10, 13].

### Limitations of the study

The limitations of this study include the different intervals of chest X-ray between subjects, so that the distribution of data is not uniform. In addition, this study has not included the effect of medication received by the patient on disease progression and chest radiography.

## Conclusions

This study proves that there is a significant relationship between the RSI chest X-ray value and the CURB-65 value with the incidence of death in hospitalized patients with COVID-19 pneumonia. RSI assessment on chest X-ray and CURB-65 values ​​are expected to assist clinicians in initial screening and planning the management of COVID-19 pneumonia patients. The combination of CURB-65 and RSI gave good results as a predictor of mortality in COVID-19 pneumonia patients.

## Data Availability

The data sets used and/or analysed during the current study are available from the corresponding author on reasonable request.

## Abbreviations

COVID-19: Coronavirus disease 2019
SARS-CoV-2: Severe acute respiratory syndrome coronavirus-2
RSI: Radiologic severity index
ROC: Receiver operating characteristic
AUC: Area under the curve
PR: Prevalence interval
